# Experimental Research on Mechanical Properties of Carbon Fiber-Reinforced Reactive Powder Concrete after Exposure to Cryogenic Temperatures

**DOI:** 10.3390/ma15124240

**Published:** 2022-06-15

**Authors:** Li Wang, Donghui Cheng, Xiaoting Wang

**Affiliations:** 1 School of Civil Engineering, Northeast Forestry University, Harbin 150038, China; wldxy@nefu.edu.cn (L.W.); nefuwxt96@nefu.edu.cn (X.W.); 2 School of Architecture and Civil Engineering, Qiqihar University, Qiqihar 161006, China

**Keywords:** reactive powder concrete, carbon fiber, cryogenic temperature, mechanical properties, stress–strain relationship

## Abstract

This study aims to evaluate the mechanical properties of carbon fiber-reinforced reactive powder concrete (CFRPC) after exposure to cryogenic temperature. The mechanical properties of plain RPC and CFRPC with carbon fiber volume contents of 0, 0.5%, 1.0%, and 1.5% were examined after exposure to 20 °C, −5 °C, −15 °C, and −25 °C for 72 h. The effect of fiber contents and exposure temperatures on the cubic and axial compressive strength, splitting tensile strength, elastic modulus, and peak strain were systematically reported and analyzed. The results showed adding carbon fiber to RPC could significantly enhance the strength and slightly improve ductility performance. Additionally, CFRPC with 1.0% fiber content showed the best mechanical properties. The maximum increases in cubic and axial compressive strength and tensile strength were 26.0%, 25.7%, and 21.8%, the elastic modulus was 13.2%, and the peak strain was 13.0% over the plain RPC. Additionally, all mechanical properties continued to degrade with decreasing temperature. After exposure to −25 °C, the cubic, axial compressive strength, and tensile strength of CFRPC degraded to 82.2–84.9%, 80.7–87.5%, and 72.7–73.7% of the normal temperature strength, respectively. In addition, the linear relationship equation between the discount factor of each mechanical property and the temperature was established. Finally, the equation for the stress–strain ascending curve of CFRPC described by a quadratic polynomial was proposed, which fitted well with the experimental results.

## 1. Introduction

The cryogenic temperature, one of the most typical extreme environments, will directly threaten the safety of concrete structures in service and lead to premature deterioration of the material. According to GB50176-2016 [1], extreme cold zones are mostly found in Northeast China, Inner Mongolia, Northern Xinjiang, Northern Tibet, and Qinghai. The 30-year average annual low temperature in January in Harbin, Heilongjiang, according to meteorological data, was −24 °C. Temperatures in Jilin, Inner Mongolia, and Xinjiang provinces were −19.9 °C, −16.9 °C, and −16.7 °C, respectively. However, the current research mostly focuses on the performance study of cryogenic concrete as liquefied natural gas storage tanks at ultra-low temperatures (T ≤ −100 °C) [2,3,4,5,6,7,8,9], while the evaluation of concrete behavior at common cryogenic temperatures (−30 °C ≤ T ≤ 0 °C) is rare. Therefore, it is important to systematically carry out the study of concrete performance at common cryogenic temperatures for the design use of structures in severe cold regions.

Nowadays, reactive powder concrete (RPC) is widely used because of its good mechanical properties and durability [10,11,12,13]. Adding fibers to RPC has been shown to greatly improve its ductility, inhibit cracking, and increase tensile strength [14,15,16,17]. Steel fiber (SF) is one of the most extensively utilized reinforcement materials among all fibers. SF can significantly improve the microstructure of RPC and enhance its tensile and compressive strength at normal temperatures, according to Zhang, Y. [18]. The mechanical properties of RPC after exposure to high temperatures were examined by Zheng, W. et al. [19]. The findings confirmed that 2% steel fibers not only prevented RPC from spalling at elevated temperatures, but also greatly improved RPC’s compressive and tensile strengths at both normal and elevated temperatures. Furthermore, after being exposed to high temperatures, compressive stress–strain equations for SFRPC were established [20]. On the other hand, Shin, W. et al. [21] confirmed that severe SF corrosion deteriorated the tensile strength of pre-cracked RPC. Aside from SF, polypropylene fiber (PPF) is also a widely utilized material in the construction industry. Canbaz, M. et al. [22] discovered that PPF alone lowered the compressive strength of RPC by 35% at normal temperature and more than 50% at 900 °C. In contrast, the research results of Zhong, C. et al. [23] indicated that PPF alone could improve the ductility, compressive strength, and splitting tensile strength of RPC. However, mixing SF and PPF is more effective, and the recommended content is 0.15% PPF + 1.75% SF. In addition, Mao, Z. et al. [24] found that RPC with 2% SF + 0.15% PPF had stronger anti-spalling and mechanical properties after exposure to elevated temperatures.

Therefore, it is necessary to develop other fiber-reinforced materials, considering that SF has high self-weight, poor corrosion resistance [25,26], and poor insulation [27,28], while PPF, although lightweight, corrosion-resistant and insulating, will degrade the mechanical properties [22]. Compared with SF and PPF, carbon fiber (CF) is a reinforcing material with significant advantages, which has the properties of lightweight and high strength, high elastic modulus, and high corrosion resistance [29,30,31,32]. The study by Liu, B. et al. [33] showed that CF had a strong bond with the concrete matrix, which could significantly improve the concrete toughness, strength, and ductility. Raza, S.S. et al. [34] also stated that CF-reinforced RPC outperforms single-doped SF and PPF. Furthermore, he demonstrated that CFRPC’s compressive strength per unit weight was much higher than SFRPC’s [35]. According to Ke, K. et al. [36], CF successfully enhanced compressive strength and other mechanical properties of RPC with an optimal volume content of 1.0%. On the other hand, Gou, J. [37] claimed that the compressive strength of RPC is greatest when the CF content is 0.5% to 1.0%. Overall, 1.5% was shown to be the most advantageous for tensile strength by Zhang, J. et al. [38]. In addition, Zhang, Y. et al. [39] discovered that CF had minimal influence on RPC’s temperature sensitivity.

Based on the current research results, it can be seen that most of the research has focused on the performance evaluation of SF and PPF reinforced RPC at room temperature and high temperature, while the exploration of the performance of CFRPC is very limited. In particular, there is a gap in the study of the performance of CFRPC after cryogenic temperatures. Therefore, tests on the mechanical properties of CFRPC with various CF volume contents after exposure to cryogenic temperatures were conducted in this work. The cubic and axial compressive strengths, splitting tensile strength, elastic modulus, and peak strain were all measured. The equation for expressing the reduction factor of each mechanical index with the temperature was derived based on experimental data. Finally, the equation for the CFRPC stress–strain ascending curve is found. As an exploratory study, the results in this paper will help to understand the mechanical behavior of CFRPC after exposure to common cryogenic temperatures and provide detailed material performance data for the structural design of CFRPC in severe cold areas.

## 2. Materials and Methods

### 2.1. Raw Materials and Mix Proportion

**Figure 1:**
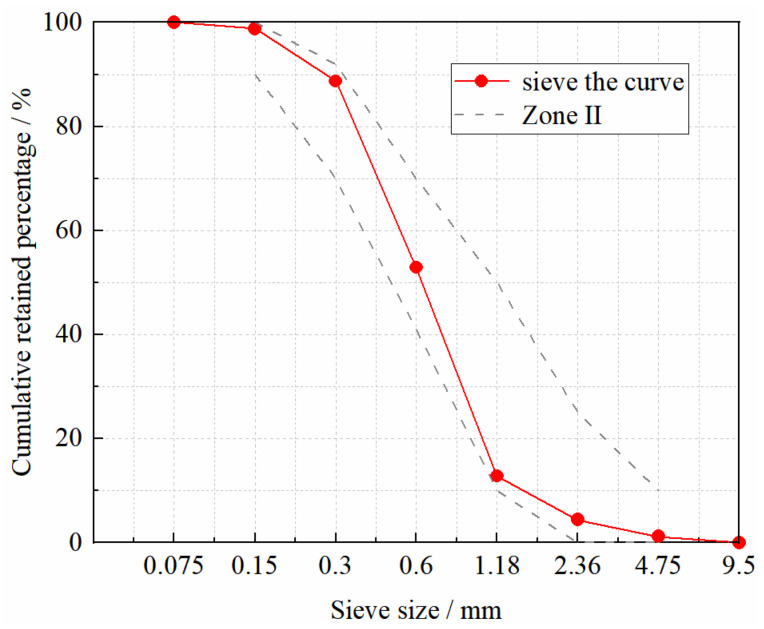
Sieve the curve for sand grading zones.

Specimens were prepared with the following ingredients: Harbin Yatai Group produces ordinary Portland cement of grade 42.5 (Chinese cement grading system). The details of cement are provided in Table 1. Borun brand silica fume was obtained, whose physical properties are shown in Table 2. Toray Ltd. in Japan produces short-cut CF. The details about CF are provided in Table 3. With a water reduction rate of 35%, Sika 530P polycarboxylate superplasticizer was used. Natural river sand was used for the fine aggregates. The sieving curve of the sand obtained from the sieving test is shown in Figure 1. The fineness modulus was calculated to be 2.53. The fine aggregate can be judged to be a well-graded Zone II medium sand. Four types of specimens were constructed, each with a CF volume content of 0, 0.5%, 1.0%, and 1.5%. Table 4 shows the mixture proportions for the test.

### 2.2. Preparation of Specimens

First, 100 mm × 100 mm × 100 mm cubic specimens were used for compressive strength testing and splitting tensile strength testing following CECS13:2009 [40]. The axial compressive strength test was performed on prismatic specimens measuring 100 mm × 100 mm × 300 mm. For each test, four groups of specimens were established based on four mix proportions corresponding to four target temperatures (20 °C, −5 °C, −15 °C, −25 °C). Each group was composed of 3 specimens, 144 specimens in total, with the arithmetic mean of 3 specimens for each group of strength determination.

**Figure 2:**
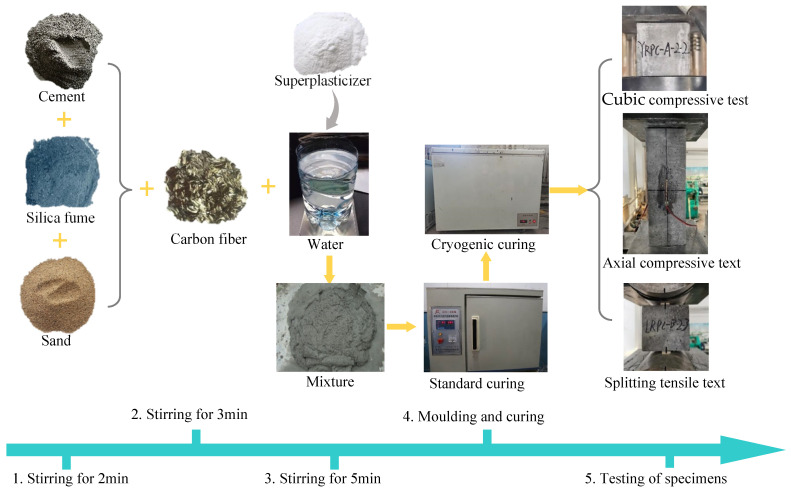
Preparation and test of process specimens.

The three-step mixing technology provided in reference [41] was adopted in the preparation of specimens to ensure a disordered uniform distribution of the CF. Figure 2 shows the preparation and test process of specimens. Firstly, the cement, silica fume, and sand were added to the concrete mixer and dry mixed for 2 min. Then, we added the CF to the mixture and stirred for 3 min. Following that, the water and superplasticizer were slowly added in the process of stirring and wet mixed for a further 5 min. Then, the mixture was injected into a steel mold, shaped by high frequency vibration on a vibrating table, and left to stand for 24 h before being demolded. The specimens were subsequently placed in the 90 °C accelerated maintenance box for 32 h, followed by 21d in the regular maintenance box. Finally, specimens were subjected to cryogenic treatment. Specimens were put in a DW-40 continuous cryogenic temperature refrigerator for curing, following the cryogenic test procedure of Miura, T. [42] and Browne, R. D. et al. [43]. The temperature targets were set at 20 °C, −5 °C, −15 °C, and −25 °C. After 72 h of cryogenic curing, the specimens were removed and restored to ambient temperature for loading testing.

### 2.3. Testing of Specimens

As shown in Figure 2, compressive tests were all performed using 2000 kN electro-hydraulic servo testing equipment with continuous uniform loading during the test, by the Chinese standard [44]. The controlled loading rate for the compressive strength test was 0.8 MPa/s and 0.08 MPa/s for the splitting tensile strength test. The resistance strain gauge BX120-50AA was used to measure the axial strain of the prismatic specimen. During the test, strains are collected at 10 kN intervals by the DH3818 static strain test system until the specimen is destroyed.

## 3. Results and Discussion

### 3.1. Cube Compressive Strength

#### 3.1.1. Compression Failure Modes of Cubic Specimens

**Figure 3:**
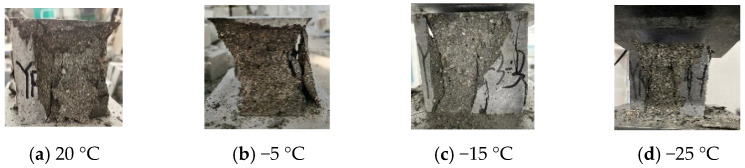
Compression failure modes of CFRPC3 cubic specimens after different exposure temperatures.

**Figure 4:**
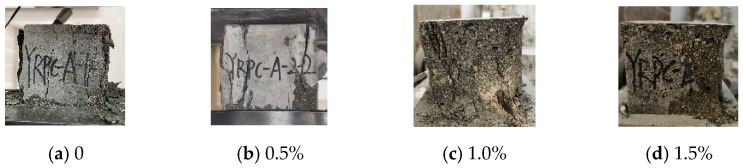
Compression failure modes of cubic specimens with different fiber contents at normal temperature.

Figure 3 shows compression failure modes of CFRPC3 cubic specimens after different exposure temperatures. As shown in Figure 3, all specimens are typical of brittle failure. Several fissures quickly grew into vertical cracks, and the cone fragments spilled in all directions, accompanied by a loud noise. The lower the temperature, the worse the specimen’s integrity at failure and the more pronounced the brittle failure features. Figure 4 depicts the failure modes of specimens with varying fiber contents at normal temperature. The brittle failure occurred in both the plain RPC and the CFRPC with 0.5% fiber content. However, for specimens with 1.0% CF and 1.5% CF, the cracks progressed slowly during loading. The specimen showed good integrity without apparent bursting at failure. The pulled CF filaments and the concrete fragments stretched by them were visible.

#### 3.1.2. Analysis of Cube Compressive Strength Results

To describe the deterioration degree of the cubic compressive strength with temperature, a coefficient *η*_cu,T_ is introduced.

(1) $$\eta_{\mathrm{cu},T}=\frac{f_{\mathrm{cu},T}}{\eta_{\mathrm{cu},20}}$$

where, *η*_cu,T_ is the cubic compressive strength reduction factor. *f*_cu,T_ is the cubic compressive strength after exposure to cryogenic temperature *T*. *f*_cu,20_ is the cubic compressive strength at 20 °C. *η*_cu,T_ ≤ 1 represents the degradation degree of cubic compressive strength after exposure to cryogenic temperature *T*.

**Figure 5:**
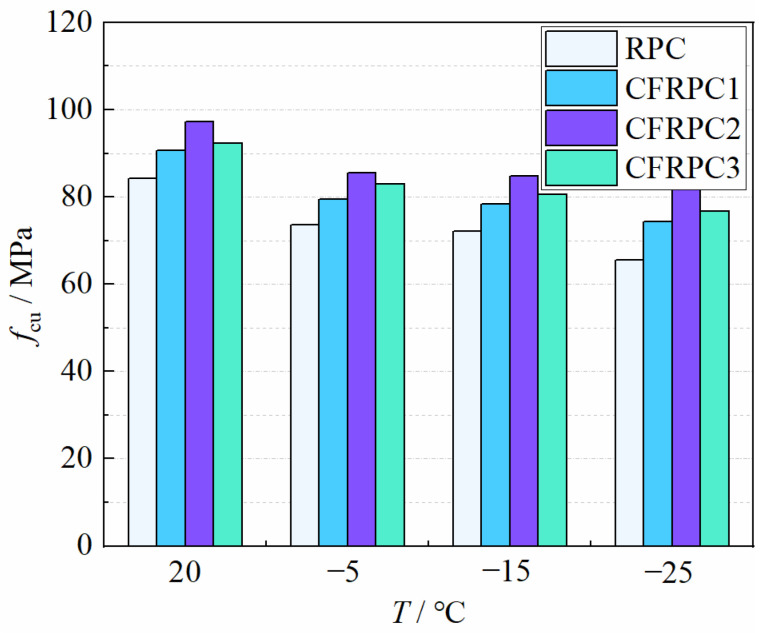
Relationship *f*_cu_ vs. *T*.

**Figure 6:**
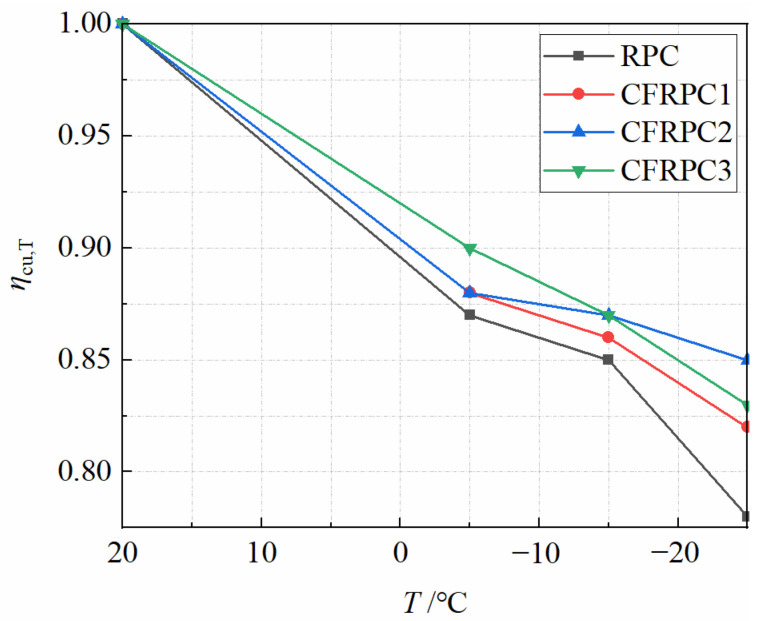
Relationship *η*_cu,T_ vs. *T*.

Figure 5 depicts the cubic compressive strengths *f*_cu_ after various exposure temperatures. Figure 6 shows the variation curves of *η*_cu,T_ with temperatures. After exposure to cryogenic temperatures, the cubic compressive strength continued to degrade with decreasing temperature. When compared to 20 °C, the strength of −5 °C, −15 °C, and −25 °C reduced by 10.2–12.7%, 15.1–22.3%, and 12.8–14.4%, respectively. Meanwhile, the plain RPC strength was more severely degraded than CFRPC. After exposure to −25 °C, the plain RPC showed the lowest strength of 65.5 MPa, which was 77.7% of the strength at 20 °C. While for CFRPC1, CFRPC2 and CFRPC3, the strengths were 74.3 MPa, 82.5 MPa, and 76.8 MPa, respectively, reducing to 82.2%, 84.9%, and 83.2% of the normal temperature strength. Based on two considerations, microstructural degeneration is the root cause of its strength degradation [45]. Firstly, according to hydraulic theory, the phase change and migration of capillary pore water in saturated concrete produce tensile stresses in pore walls at cryogenic temperatures. When the tensile stress reaches the ultimate tensile strength of concrete, the pores shatter and microcracks appear. Second, concrete is a non-homogeneous material with different thermal expansion coefficients for each component ingredient. The high internal stresses generated at each component’s contact during the cooling process cause further structural damage.

On the other hand, CF addition significantly improved the cubic compressive strength of RPC after different exposure temperatures. CFRPC1, CFRPC2 and CFRPC3 showed 7.5–13.4%, 15.3–26.0% and 9.5–17.3% higher than the plain RPC, respectively. This is due to the high tensile strength and elastic modulus of CF. CF that are evenly distributed in the concrete can be bonded with the matrix to generate a network structure that improves stiffness and toughness. CF can restrain the initial cracks and microcracks in the specimen, inhibiting premature cracking and allowing the strength of the base material to be fully utilized. However, due to their thin and short length, the CF cannot prevent the formation and development of large cracks. This is the main reason why CF-reinforced RPC specimens still exhibit brittle characteristics at failure. In addition, CFRPC2 with 1.0% CF showed the best cubic compressive strength. From 20 °C to −25 °C, the strength was 97.2 MPa, 85.6 MPa, 84.7 MPa, and 82.5 MPa, respectively, which was 15.3%, 16.3%, and 17.3%, and 26.0% higher than the plain RPC. When the CF content is less than 1.0%, the fracture resistance and strength enhancement of fibers is not obvious, and the specimen strength is poor. When the CF content was more than 1.5%, due to the excessive fibers produced by the “pellet effect”, the formation of weak interface areas, increasing the internal defects of concrete, which in turn leads to an unsatisfactory reinforcement effect. As a result, 1.0% is advised as the appropriate CF volume content, which is consistent with the reference [36].

**Figure 7:**
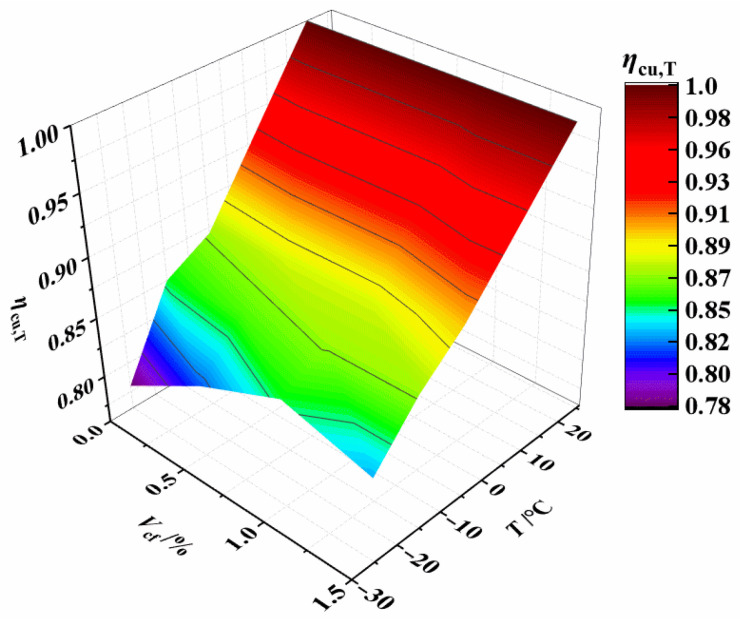
Relationship *η*_cu,T_ vs. *T* and *V*_cf_.

The effect of temperature and CF content on *η*_cu,T_ can be more visually expressed in Figure 7. It is clear that temperature has a more significant effect on *η*_cu,T_ than CF content. For broad application, a linear function is utilized to describe the relationship between *η*_cu,T_ and *T*, as expressed by Equation (2). *R*^2^ = 0.965, the linear fitting formula is highly accurate.

(2) $$\eta_{\mathrm{cu},T}=3.71\left(\frac{T}{1000}\right)+0.92\left(R^{2}=0.965\right)\;\;-25\;{}^{\circ}C\leq T\leq 20\;{}^{\circ}C$$

### 3.2. Axial Compressive Strength

#### 3.2.1. Compression Failure Modes of Prismatic Specimens

**Figure 8:**
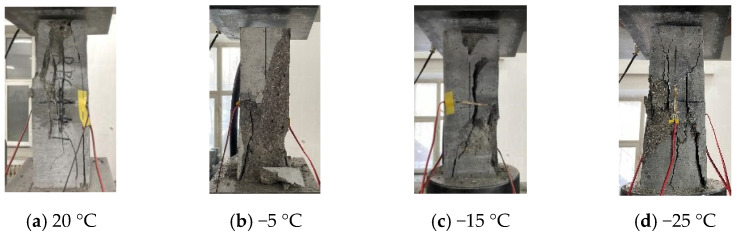
Compression failure modes of CFRPC3 prismatic specimens after different exposure temperatures.

Figure 8 shows the failure modes of CFRPC3 specimens after various exposure temperatures. Shear failure, tensile failure, and composite failure were all possible failure modes [46]. At normal temperature, the prismatic failure mode consisted mostly of tensile and shear failure. Tensile failure was shown by multiple vertical major fractures running through the section, the breadth of the cracks was significant, and the specimen was divided into several prisms, accompanied by a loud bursting sound. Shear failure was defined as a diagonal crack through the specimen’s corner, the aggregate splitting, CF sheared off, and the concrete cone being shattered and falling off, with a slight cracking sound. The axial compression failure of the prismatic specimen following cryogenic temperatures, on the other hand, was a composite failure mode. The specimens’ two ends had oblique cracks that stretched to the center and formed two intersecting cracks. Additionally, one end of the specimen was crushed and shattered into a triangular cone. Furthermore, the lower the temperature, the poorer the specimen’s integrity at failure.

**Figure 9:**
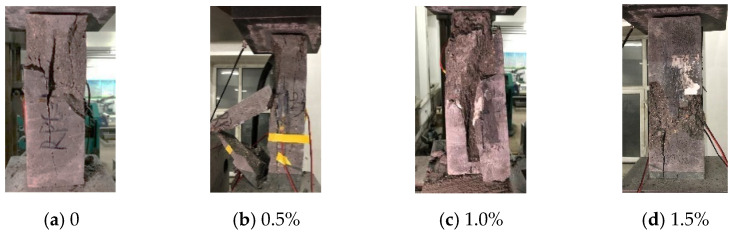
Compression failure modes of prismatic specimens with different fiber contents at normal temperature.

The addition of CF changes the failure mode, as shown in Figure 9. A tensile failure occurred in the plain RPC specimens. Tensile shear failure was detected in CFRPC with fiber contents of 0.5% and 1.0%, whereas shear failure was reported at 1.5%. Therefore, as the CF content increased, the failure mode of CFRPC steadily shifted from tensile shear failure to shear failure. The top and bottom cones of the specimen with 1.5% fiber content, in particular, showed good integrity at failure, whereas the center region was divided into multiple regular prisms and made a dull sound. It showed that CF significantly improved brittle properties of CFRPC.

#### 3.2.2. Analysis of Axial Compressive Strength Results

**Figure 10:**
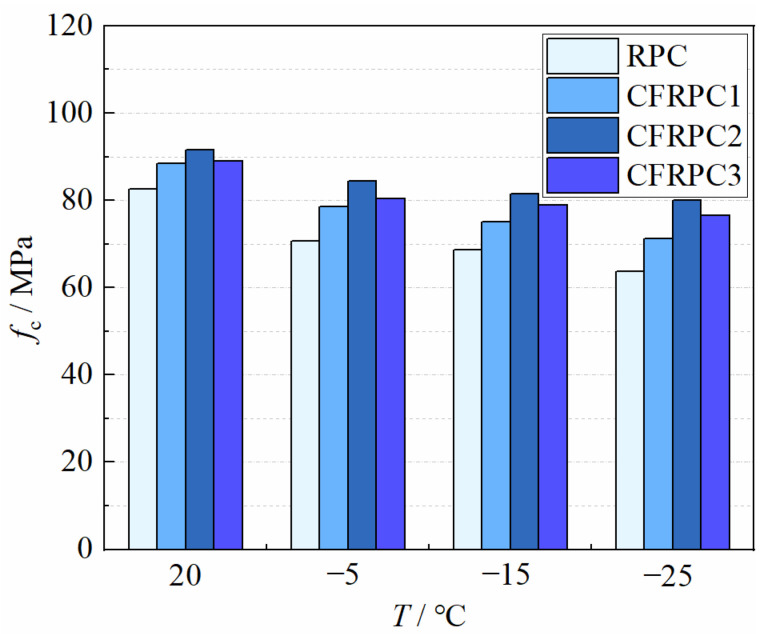
Relationship *f*_c_ vs. *T*.

**Figure 11:**
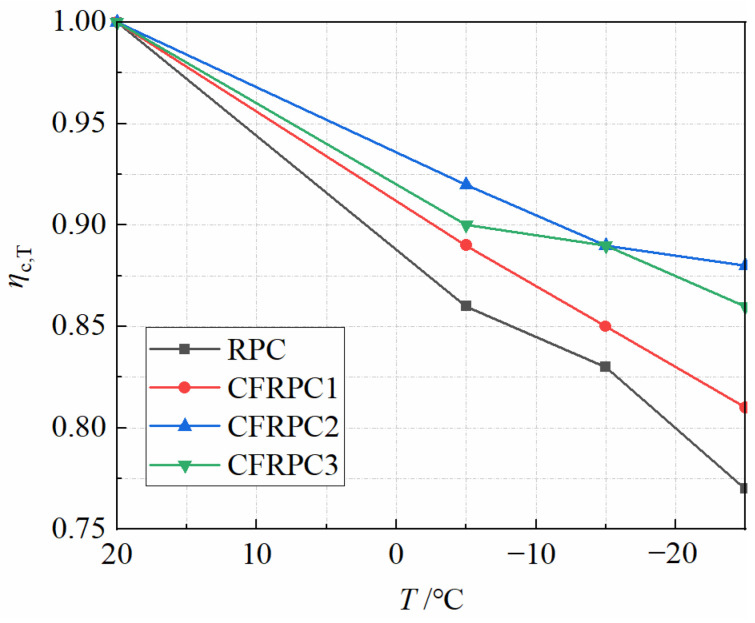
Relationship *η*_c,T_ vs. *T*.

The axial compressive strengths *f*_c_ of CFRPC after different exposure temperatures are given in Figure 10. The variation curve of *η*_c,T_ with *T* is given in Figure 11. *η*_c,T_ is the axial compressive strength reduction factor and is calculated in the same way as Equation (1). The axial compressive strength decreased as the temperature dropped. The strengths at −5 °C, −15 °C and −25 °C were 7.7–14.3%, 11.0–16.8% and 12.5–22.8% lower than at 20 °C, respectively. Meanwhile, the plain RPC strength was more severely degraded than CFRPC. After exposure to −25 °C, the plain RPC showed the lowest strength of 63.7 MPa, which was 77.2% of the strength at 20 °C. While for CFRPC1, CFRPC2 and CFRPC3, the strengths were 71.3 MPa, 80.1 MPa and 76.6 MPa, respectively, reducing to 80.7%, 87.5%, and 86.1% of the normal temperature strength. The degradation of axial compressive strength, like that of cubic compressive strength, is caused by microstructural degeneration after cryogenic temperatures. Meanwhile, CF enhanced the axial compressive strength. When compared to plain RPC, the strengths of CFRPC1, CFRPC2, and CFRPC3 were enhanced by 7.2–11.9%, 10.9–25.7%, and 7.9–20.3%, respectively. Furthermore, CFRPC2 with 1.0% CF performed better in axial compressive strength compared to other fiber content. From 20 °C to −25 °C, the strengths of CFRPC2 were 91.5 MPa, 84.5 MPa, 81.4 MPa, and 80.1 MPa, respectively, which were 10.9%, 19.5%, 18.7%, and 25.7% higher than plain RPC.

**Figure 12:**
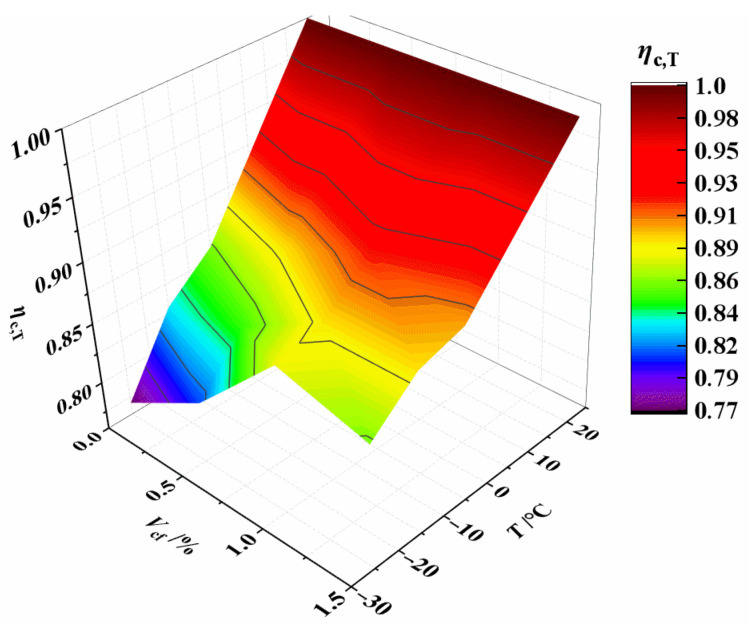
Relationship *η*_c,T_ vs. *T* and *V*_cf_.

The effect of temperature on *η*_c,T_ is more significant, as shown in Figure 12. Equation (3) is used to describe the relationship between *η*_c,T_ and *T*. The correlation coefficient is *R*^2^ = 0.911 with high accuracy.

(3) $$\eta_{c,T}=3.43\left(\frac{T}{1000}\right)+0.93\left(R^{2}=0.911\right)\;\;-25\;{}^{\circ}C\leq T\leq 20\;{}^{\circ}C$$

#### 3.2.3. The Relationship between Axial Compressive Strength and Cubic Compressive Strength

**Figure 13:**
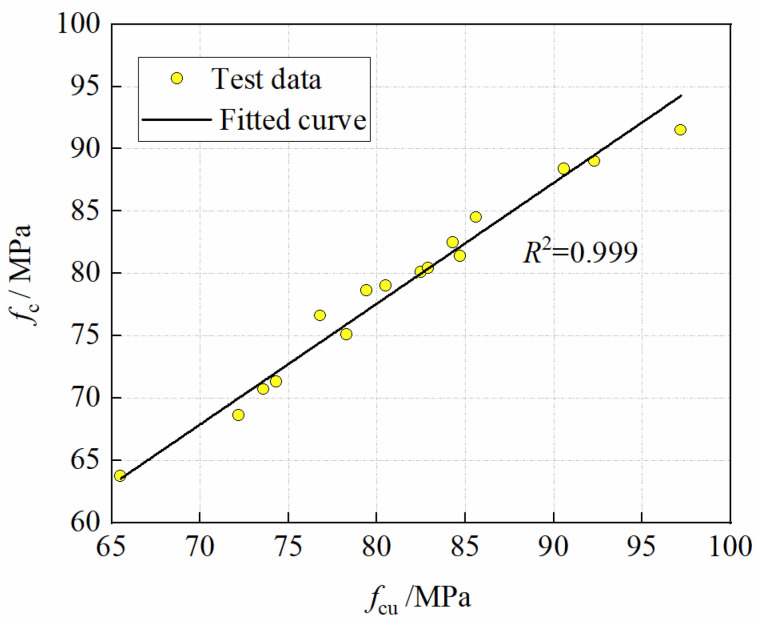
Relationship *f*_c_ vs. *f*_cu_.

As shown in Figure 13, the axial compressive strength is linearly related to the cubic compressive strength, with the linear regression yielding

(4) $$f_{c}=0.97f_{\mathrm{cu}}\;\;-25\;{}^{\circ}C\leq T\leq 20\;{}^{\circ}C$$

The ratio of axial compressive strength to cubic compressive strength is 0.97 can be obtained from Equation (4). It is much larger than 0.76 for ordinary concrete and 0.82 for high-strength concrete. It means that the higher the strength of concrete, the closer *f*_c_/*f*_cu_ is to 1.0. It is assumed that both CFRPC axial compressive strength and cubic compressive strength obey normal distribution, and the coefficient of variation of the two strengths is not significantly different by calculation. Therefore, *f*_c,k_ and *f*_cu,k_ also conform to the relationship of Equation (4).

### 3.3. Splitting Tensile Strength

#### 3.3.1. Splitting Tensile Failure Modes of Cubic Specimens

**Figure 14:**
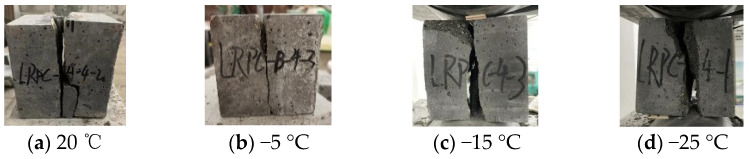
Splitting tensile failure modes of CFRPC3 cubic specimens after different exposure temperatures.

**Figure 15:**
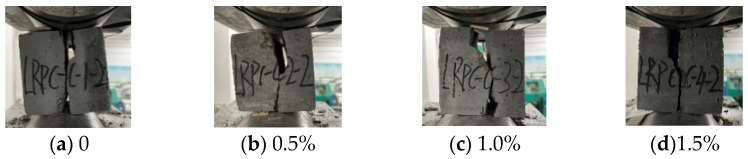
Splitting tensile failure modes of cubic specimens with different fiber contents at −15 °C.

The splitting tensile failure mode of specimens was brittle, as shown in Figure 14 and Figure 15. The failure process was similar: the specimens broke as soon as they were cracked, and there was only one main crack. The fracture profile was clear at failure, with a small amount of fragment flaking off, accompanied by a violent sound. In particular, compared with the specimens at normal temperature, the failure process of the specimens after exposure to cryogenic temperature was more abrupt, the sound was crisper, and the brittle failure characteristics were more significant.

#### 3.3.2. Analysis of Splitting Tensile Strength Results

**Figure 16:**
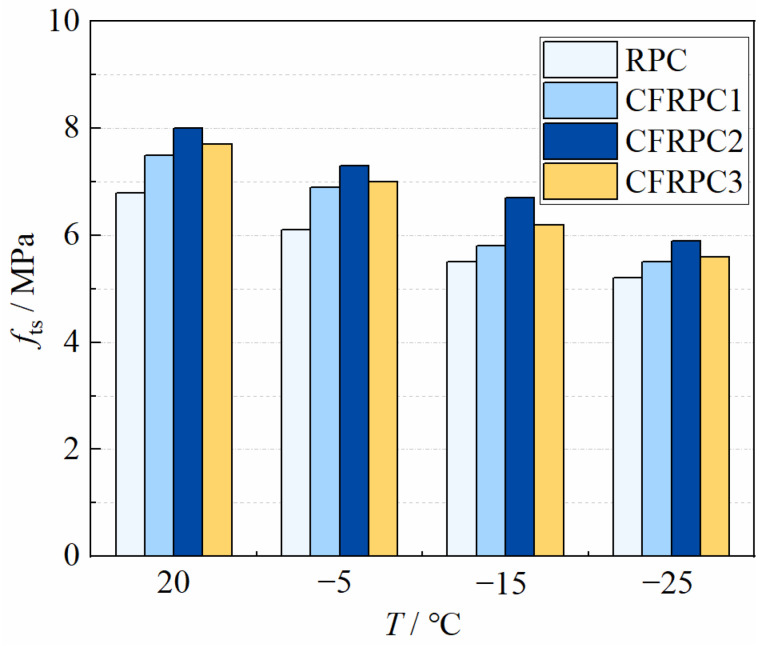
Relationship *f*_ts_ vs. *T*.

**Figure 17:**
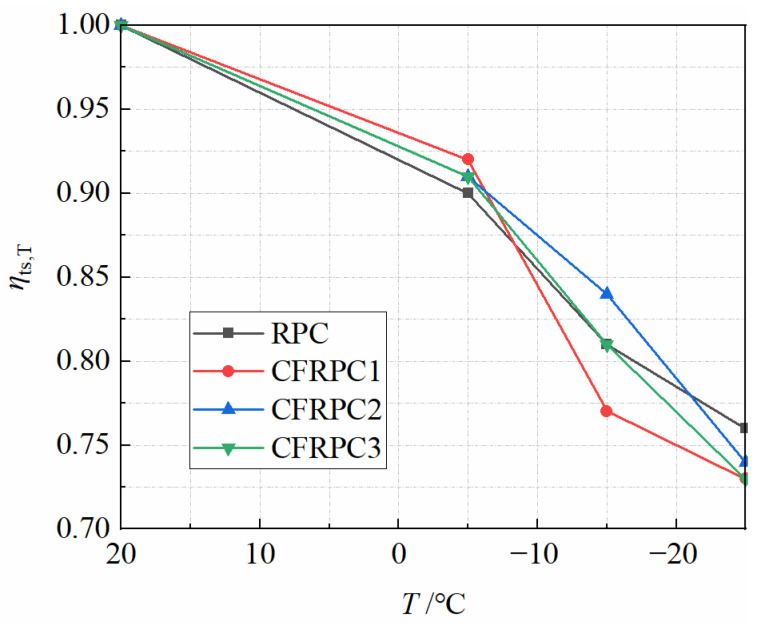
Relationship *η*_ts,T_ vs. *T*.

Tensile strength is another important mechanical property of concrete. Figure 16 depicts the splitting tensile strengths *f*_ts_ of CFRPC after various exposure temperatures. Figure 17 shows the variation curve of *η*_ts,T_ with *T*. *η*_ts,T_ is the reduction factor of splitting tensile strength and calculated in the same way as Equation (1). As with the compressive strength, the splitting tensile strength decreased significantly with the drop of temperature. The strengths at −5 °C, −15 °C, and −25 °C decreased by 8.0–10.3%, 16.3–22.7%, and 23.5–27.3%, respectively, compared to 20 °C. After exposure to −25 °C, the plain RPC had the lowest splitting tensile strength of 5.2 MPa, which was 76.5% of the strength at 20 °C. For CFRPC1, CFRPC2 and CFRPC3, the strengths were 5.5 MPa, 5.9 MPa, and 5.6 MPa, respectively, reducing to 73.3%, 73.7%, and 72.7% of 20 °C strength. The reason for large strength deterioration is the intensified cryogenic temperature damage and the continuous development of internal cracks in specimens. A comparison of the compressive strength showed that the tensile strength degraded more severely, due to a greater sensitivity to structural microcracks caused by the decrease in temperature. Moreover, CFRPC splitting tensile strength was higher than RPC after different cryogenic temperatures, indicating a positive effect of CF. CFRPC1, CFRPC2, and CFRPC3 improved by 5.5–13.1%, 13.5–21.8%, and 14.8–7.7% over the plain RPC, respectively. This is due to CF excellent crack resistance, which contributes to the ultimate tensile strength of concrete. Furthermore, CFRPC2 with 1.0% CF showed the highest splitting tensile strength. From 20 °C to −25 °C, 17.6%, 19.7%, 21.8%, and 13.5% increased, respectively.

**Figure 18:**
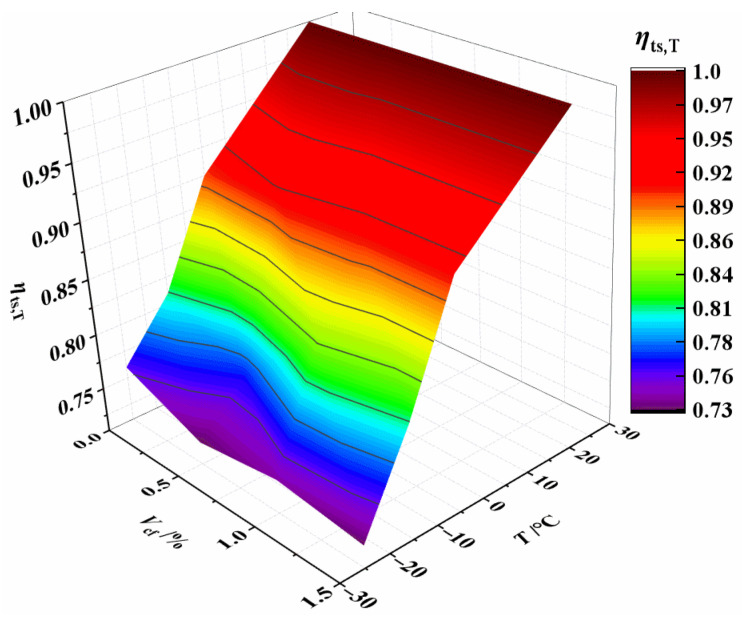
Relationship *η*_ts,T_ vs. *T* and *V*_cf_.

The contour lines from Figure 18 show that temperature has a greater impact on *η*_ts,T_ than fiber content. As a result, the linear function of Equation (5) is employed to characterize the relationship between temperatures and *η*_ts,T_. *R*^2^ = 0.920, the linear fitting formula is highly accurate.

(5) $$\eta_{\mathrm{ts},T}=5.88\left(\frac{T}{1000}\right)+0.90\left(R^{2}=0.920\right)\;\;-25\;{}^{\circ}C\leq T\leq 20\;{}^{\circ}C$$

#### 3.3.3. The Relationship between Splitting Tensile Strength and Cubic Compressive Strength

**Figure 19:**
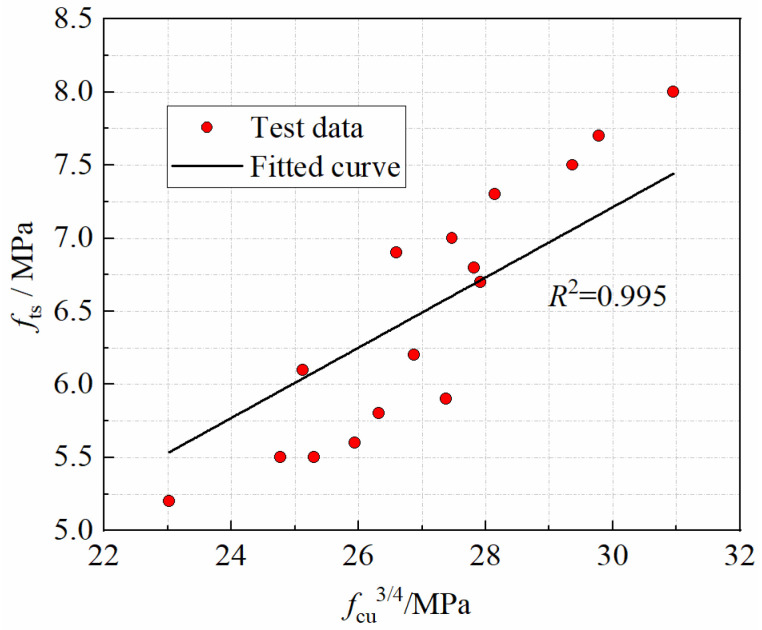
Relationship *f*_ts_ vs. *f*_cu_^3/4^.

As shown by Equation (6), the specification GB 50010-2010 [47] gives the relationship between the splitting tensile strength and the cubic compressive strength of ordinary concrete at normal temperature expressed as an exponential function. The exponential function relationship obtained for the experimental data in this paper is expressed in Equation (7). The larger value of the coefficient in Equation (7) indicates that the CF is more effective in improving the tensile strength of CFRPC. The relationship curve between splitting tensile strength and cubic compressive strength is shown in Figure 19.

(6) $$f_{\mathrm{ts}}=0.19f_{\mathrm{cu}}^{3/4}$$

(7) $$f_{\mathrm{ts}}=0.24f_{\mathrm{cu}}^{3/4}\;\;-25\;{}^{\circ}C\leq T\leq 20\;{}^{\circ}C$$

### 3.4. Elastic Modulus and Peak Strain

#### 3.4.1. Elastic Modulus

**Figure 20:**
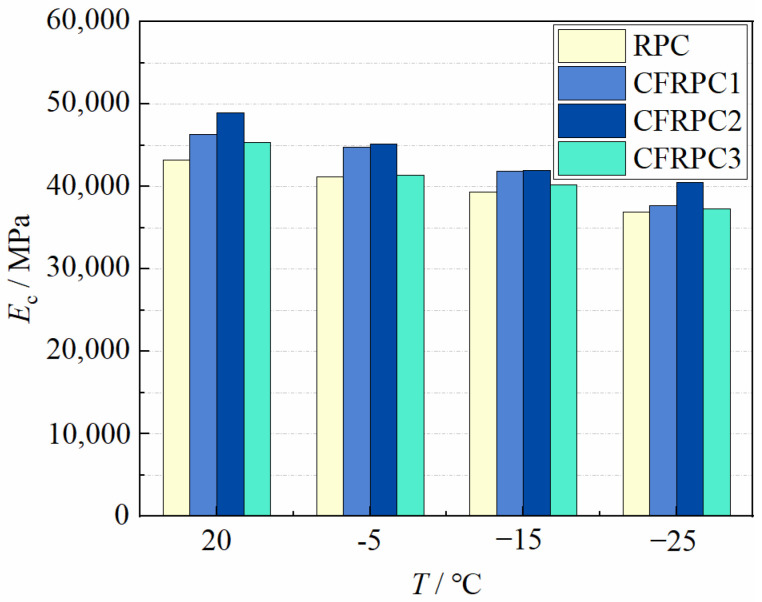
Relative *E*_c_ vs. *T*.

**Figure 21:**
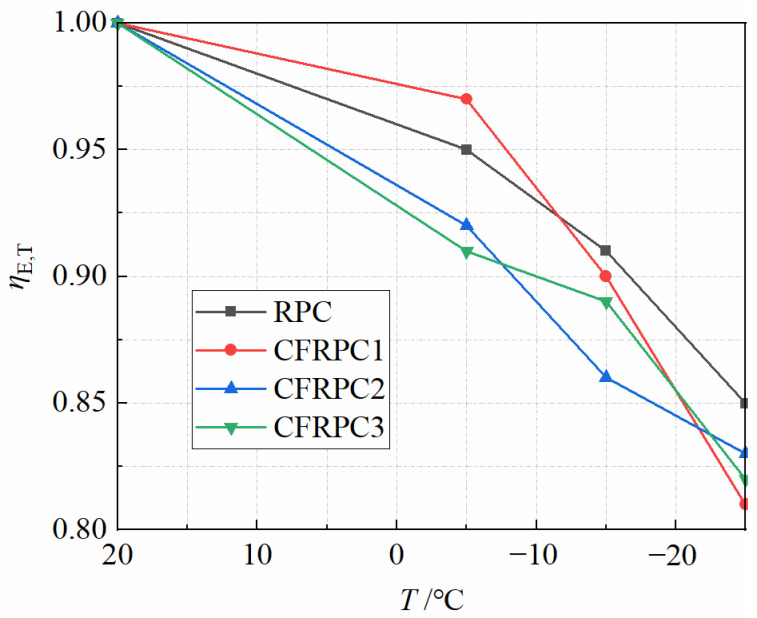
Relationship *η*_E,T_ vs. *T*.

Figure 20 depicts the elastic modulus after exposure to various temperatures. It can be seen that compared with the plain RPC, the elastic modulus of CFRPC1, CFRPC2, and CFRPC3 increased by 2.2–8.7%, 6.9–13.2%, and 0.5–4.9%, respectively. Additionally, the elastic moduli of RPC and CFRPC at room temperature are 43,200 MPa and 45,300–48,900 MPa, respectively, which are much higher than that of high-performance concrete and ordinary concrete [48]. However, after exposure to low temperatures, the elastic modulus at −5 °C, −15 °C, and −25 °C decreases by 3.2–8.6%, 9.0–14.1% and 14.6–18.6% over that of 20 °C (Figure 21), respectively. This indicates that, in the same way as strength, low temperatures cause degradation of elastic modulus. Therefore, *η*_E,T_ is defined as the elastic modulus reduction factor and calculated in the same way as Equation (1). Additionally, the relationship between *η*_E,T_ and *T* is described by Equation (8), which fitted the test results well.

(8) $$\eta_{E,T}=3.85\left(\frac{T}{1000}\right)+0.93\;\left(R^{2}=0.920\right)\;\;-25\;{}^{\circ}C\leq T\leq 20\;{}^{\circ}C$$

From the experimental results of this paper and references [49,50], it can be seen that the elastic modulus increases with the increase of strength, but they are not directly proportional. Based on the test data, the relationship between the elastic modulus *E*_c_ and the axial compressive strength *f*_c_ of CFRPC after exposure to cryogenic temperatures can be obtained by non-linear regression as follows

(9) $$E_{c}=4735\sqrt{f_{c}}\left(R^{2}=0.997\right)\;\;-25\;{}^{\circ}C\leq T\leq 20\;{}^{\circ}C$$

(10) $$E_{c}=\left(0.2914\sqrt{f_{c}}+1.041\right)\times 10^{4}$$

(11) $$E_{c}=3027\sqrt{f_{c}}+9533$$

**Figure 22:**
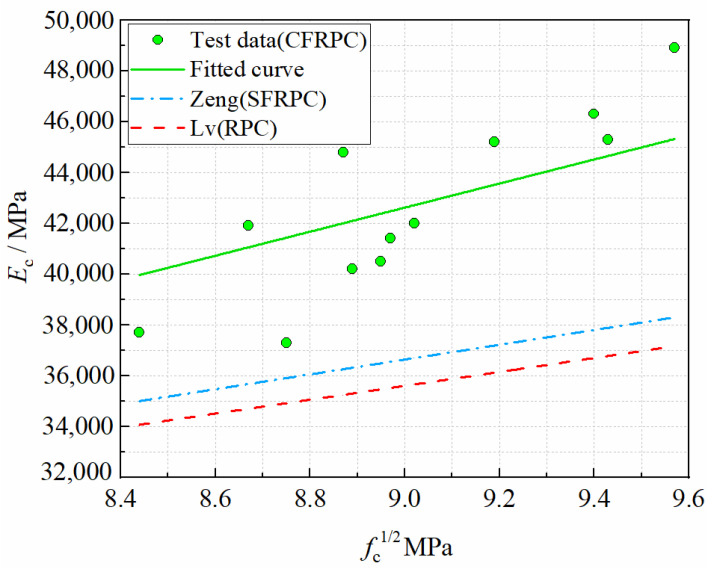
Relationship *E*_c_ vs. *f*_c_.

Equations (10) and (11) are the fitted equations for the elastic moduli of SFRPC [49] and RPC [50] at room temperature, respectively. A comparison between the predicted values and the fitted curves of Equation (9) is shown in Figure 22. It is obvious that both predicted values are conservative for the elastic modulus of CFRPC. The fitted curve of Equation (9), on the other hand, agrees better with test results, and its trend is similar to that of SFRPC and plain RPC at room temperature. Therefore, it is recommended that Equation (9) be used to calculate the elastic modulus of CFRPC after exposure to cryogenic temperatures.

#### 3.4.2. Peak Strain

**Figure 23:**
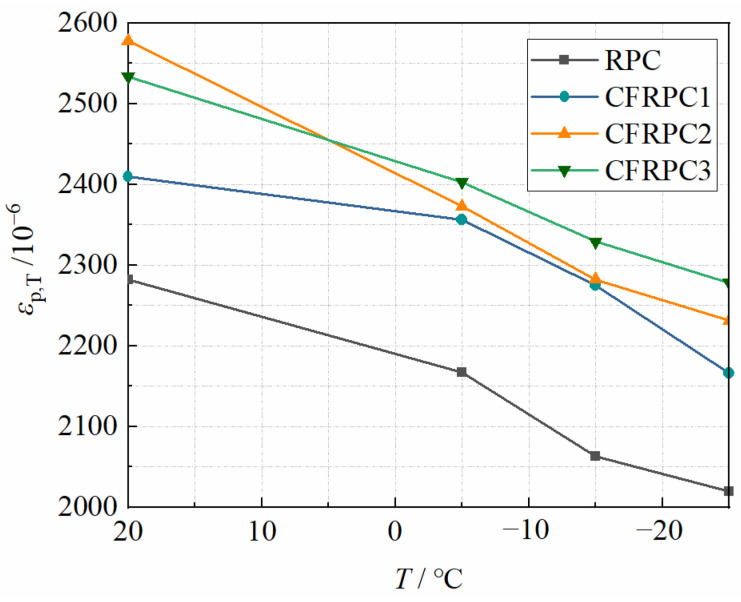
Relationship *ε*_p__,T_ vs. *T*.

**Figure 24:**
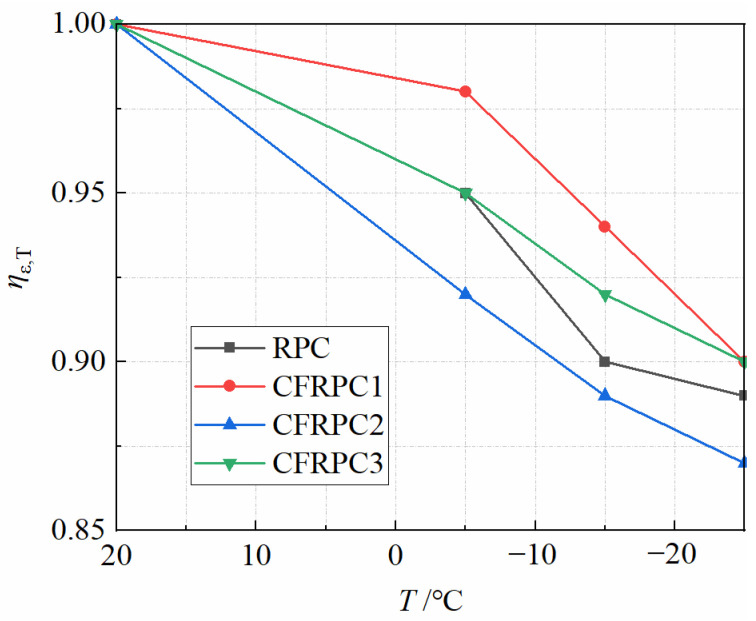
Relationship *η*_ε,T_ vs. *T*.

Peak strain, to some extent, indicates concrete ductility. In general, the higher the peak strain, the greater the material’s ductility [51]. It can be seen from Figure 23 that the peak strains of CFRPC1, CFRPC2, and CFRPC3 increase by 5.6–10.3%, 9.5–13.0%, and 10.9–12.9% compared to the plain RPC. This is because CF can help to delay the crack formation and acts as a bridge in existing cracks, improving the ductility of CFRPC to some extent. However, the comparison with strength shows that CF contributes less to ductility performance. Besides, the general trend of the curves in Figure 23 shows that the peak strain decreases gradually with decreasing temperature. The peak strains at −5 °C, −15 °C and −25 °C decrease by 2.2–8.0%, 5.6–11.5% and 10.1–13.4%, respectively, compared to 20 °C, as shown in Figure 24. This indicates that low temperatures have a greater negative effect on ductility. Therefore, *η*_ε,T_ is introduced as a peak strain reduction factor to express the degradation of peak strain due to low temperature, calculated in the same way as Equation (1). Additionally, the linear relationship between *η*_ε,T_ and *T* is expressed as follows:

(12) $$\eta_{\varepsilon ,T}=2.43\left(\frac{T}{1000}\right)+0.95\left(R^{2}=0.877\right)\;\;-25\;{}^{\circ}C\leq T\leq 20\;{}^{\circ}C$$

**Figure 25:**
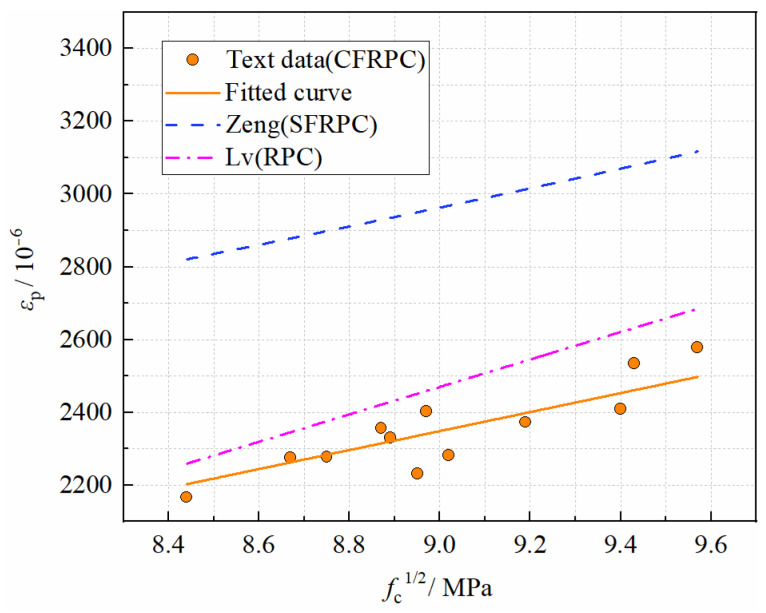
Relationship *ε*_p_ vs. *f*_c_.

As shown in Figure 25, the peak strain also increases with strength. By regression analysis of the measured data in this paper, the relationship between the peak strain and the axial compressive strength of CFRPC after exposure to cryogenic temperatures can be established as:

(13) $$\varepsilon_{p}=261\times 10^{-6}\sqrt{f_{c}}\left(R^{2}=0.994\right)\;\;-25\;{}^{\circ}C\leq T\leq 20\;{}^{\circ}C$$

The curves of the peak strain of SFRPC [49], RPC [50] at normal temperatures are also given in Figure 25. By comparative analysis, it is found that both predicted values are much higher than the measured test values, which is inappropriate. Considering the differences in fiber type and temperature, it is recommended that Equation (13) be used to calculate the peak strain of CFRPC after cryogenic temperatures. In addition, the peak strain of SFRPC is greater than that of CFRPC, indicating that SF may be more desirable for ductility improvement of RPC.

### 3.5. Stress–Strain Curves

#### 3.5.1. Measured Compressive Stress–Strain Ascending Curves

**Figure 26:**
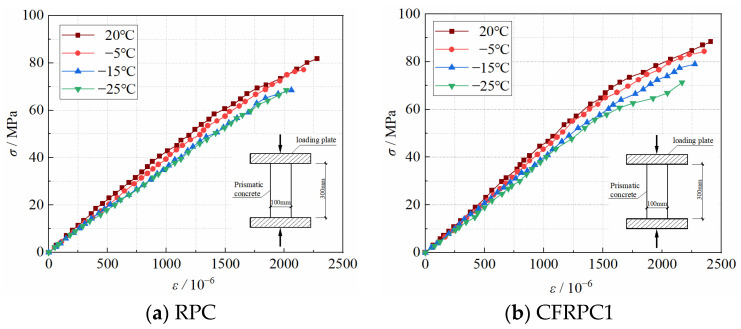

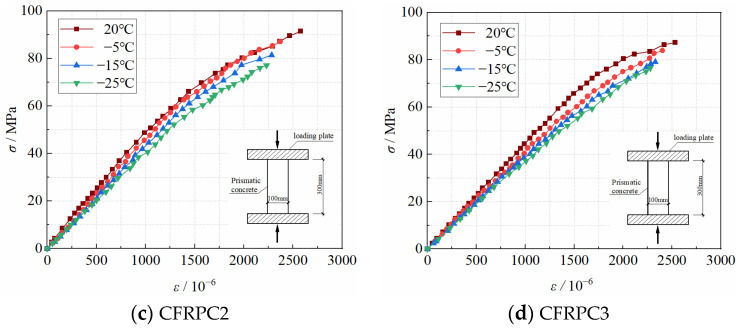
Stress–strain curves of CFRPC after different exposure temperatures.

Figure 26 shows the stress–strain ascending curves of CFRPC after exposure to various temperatures. Due to the low stiffness of the loading device, the energy inside the specimen is rapidly released after the ultimate load is reached, so that the ideal descending curve cannot be obtained. The stress–strain curves of CFRPC following exposure to cryogenic temperature exhibit comparable geometric properties and change patterns. Each curve tends to vary linearly during the loading process, and the specimen essentially retains linear elasticity. The stress–strain curve following cryogenic temperature has the same shape as the normal temperature curve at the beginning of the loading process. As the temperature drops, the microstructure damage in concrete worsens, and the strength and deformation modulus continue to deteriorate. The stress–strain curve’s ultimate stress and peak strain steadily diminish, and the peak point is moved right and down. The specimens are destroyed as soon as the ultimate stress is attained, indicating a high degree of brittleness.

**Figure 27:**
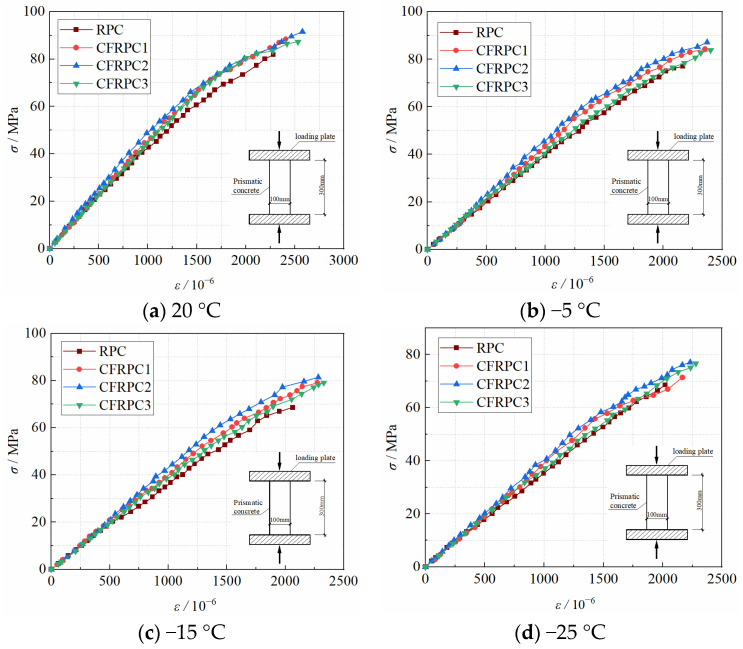
Stress–strain curves of CFRPC with different CF contents.

Figure 27 compares the stress–strain curves of CFRPC with different fiber contents following exposure to different temperatures. It can be observed that during the loading process, each curve trend is essentially the same and tends to be linear. The stress–strain curve of CFRPC is flatter than that of plain RPC, the peak point shifts right and up, and the ultimate stress and peak strain rise. It shows that adding CF may effectively improve the brittle characteristics of concrete, raise the peak strain, and improve the mechanical properties of CFRPC under axial pressure. It is worth noticing, in particular, that the stress–strain curve of CFRPC2 exhibits the flattest trend, with the highest ultimate stress and peak strain at the same temperature. It shows that at 1.0% fiber content, the greatest strength, and ductility are reached.

#### 3.5.2. The Equation for Compressive Stress–Strain Ascending Curve

Polynomials are mostly used to express the equation of the stress–strain ascending curves for RPC and fiber-reinforced RPC, as shown in Table 5. Therefore, based on the characteristics of the stress–strain curve (Figure 26 and Figure 27), a quadratic polynomial expression was proposed to express the stress–strain equation in this paper. To facilitate analysis, dimensionless coordinates were used to develop a unified stress–strain equation for varied temperatures and CF volume contents. The stress–strain curves are transformed into standard curves with abscissa *x* = *ε*/*ε*_p,T_ and ordinate *y* = *σ*/*σ*_p,T_, where *ε* and *σ* denote stress and strain, and *ε*_p,T_ and *σ*_p,T_ denote peak strain and peak stress after cryogenic temperature. CFRPC stress–strain curves after various cryogenic temperatures had the same geometric properties as those at normal temperatures. Additionally, both of them may be described by the same equation. Therefore, the compressive stress–strain equation for an ascending curve was fitted with a quadratic polynomial, as follows:

*y* = *α*_1_*x* + *α*_2_*x*^2^ (14)

Taking the boundary constraints *x* = 0 and *y* = 0 into account, *α =* 0.

With the boundary constraints *x* = 1 and *y* = 1, we obtain

*α*_1_ + *α*_2_ = 1 (15)

Noting *α*_1_ as *α*, the equation is obtained

*y* = *αx* + (1 − *α*) *x*^2^ (16)

It is clear from Equation (16) that different curves can be formed for different values of α.

When *x* = 0, *α* is as follows:

(17) $$\alpha ={\frac{dy}{dx}|}_{x=0}={\frac{d\left(\sigma /\sigma_{p,T}\right)}{d\left(\varepsilon /\varepsilon_{p,T}\right)}|}_{x=0}=\frac{{d\sigma /d\varepsilon |}_{x=0}}{\sigma_{p,T}/\varepsilon_{p,T}}=\frac{E_{0,T}}{E_{P,T}}$$

where the physical meaning of α is the ratio of the tangential elastic modulus, *E*_0,T_ at the curve’s zero point to the secant modulus, *E*_P,T_ at the curve’s highest point. From the shape of stress–strain curves measured in tests, it is clear that *E*_0,T_ ≥ *E*_P,T_, so *α* ≥ 1.

**Figure 28:**
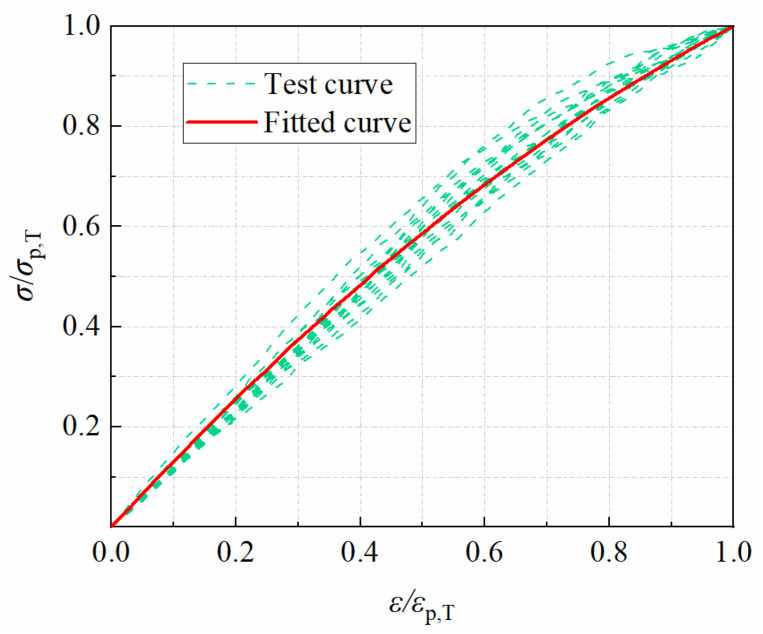
Comparison of test curve and fitted curve of stress–strain.

Based on the findings of the tests in this study, *α* = 1.35 is taken and replaced into Equation (16). As illustrated in Equation (18), the equation for the compressive stress–strain curve of CFRPC after cryogenic temperatures is found. In Figure 28, the fitted curve is compared to the experimental data. The normalized stress–strain fitted curve of CFRPC is well matched with the test curve.

*y* = 1.35*x* + (1 − 0.35)*x*^2^  0 ≤ *x* ≤ 1 (18)

## 4. Conclusions

In this study, the mechanical properties of CFRPC after exposure to cryogenic temperatures were investigated. The following conclusions can be drawn from the experimental results:

1. Adding CF to RPC can significantly enhance its strength and slightly improve ductility performance. CFRPC with 1.0% fiber volume content showed the best mechanical properties. The maximum increase in cubic and axial compressive strength and tensile strength is 26.0%, 25.7%, 21.8%, the elastic modulus is 13.2%, and the peak strain is 13.0% over the plain RPC.
2. After exposure to cryogenic temperatures, strength and ductility continued to degrade with decreasing temperature. After exposure to −25 °C, the plain RPC showed 77.7%, 77.2%, and 76.5% lower cubic, axial compressive strength, and tensile strength than those of the normal temperature, respectively. While for CFRPC, the cubic, axial compression, and tensile strength degraded to 82.2–84.9%, 80.7–87.5%, and 72.7–73.7% of normal temperature strength, respectively.
3. Equations to express the linear relationship between the discount factor of cubic compressive strength, axial compressive strength, splitting tensile strength, elastic modulus, and peak strain with the exposure temperature were established in this paper. Moreover, the linear relationship between *f*_c_ and *f*_cu_, the exponential function relationship between *f*_ts_ and *f*_cu_, and the root function relationship between *E*_c_ and *f*_c_, *ε*_p_ and *f*_c_ were defined. Each equation agreed well with the test data. The mechanical properties and their relationship equations in this study can provide a basis for the research and engineering application of CFRPC in severe cold regions.
4. The compressive stress–strain ascending curves of CFRPC with different CF contents and after different cryogenic temperatures showed similar linear characteristics. Comparative analysis showed that, as the temperature decreased, the peak point shifted to the right and downwards, and the ultimate stress, peak strain gradually decreased. Cryogenic temperatures caused degradation of the strength and ductility of CFRPC. Finally, a quadratic polynomial equation expressing the stress–strain ascending curve was proposed for CFRPC at room temperature and after exposure to cryogenic temperatures, which fitted the test results well.

## Figures and Tables

**Table 1:** Physical properties of cement.

Name	Density/g·cm^−3^	Fineness/%	Standard Consistency Water Consumption/%	Setting Time/min		Compressive Strength/MPa		Stability
				Initial Setting Time	Final Setting Time	3d	28d	
Cement	1.5	1.2	26.0	190 min	270 min	17.0	46.2	Qualified

**Table 2:** Physical properties of silica fume.

Name	SO_2_ Content/%	Burning Loss/%	Density/g·cm^−3^	Specific Surface Area/m^2^g^−1^	Water Demand Ratio	Chlorine Ion Content/%	28d Activity Index/%
Silica fume	96.1	3.9	1.8	19.1	125	0.07	98.0

**Table 3:** Physical properties of CF.

Name	Diameter/mm	Length/mm	Density/g·cm^−3^	Tensile Modulus/GPa	Tensile Strength/MPa
Carbon fiber	7.0	10.0	1.8	228	4900

**Table 4:** Mixture proportions for the test.

Name	W/B	Cement/kg·m^−3^	Silica Fume/kg·m^−3^	Sand/kg·m^−3^	Water Reducer/kg·m^−3^	*V*_cf_/%
RPC	0.22	637	193	1280	15	0
CFRPC1	0.22	637	193	1280	15	0.5%
CFRPC2	0.22	637	193	1280	15	1.0%
CFRPC3	0.22	637	193	1280	15	1.5%

Note: *V*_cf_ indicates CF volume content.

**Table 5:** Stress–strain ascending curve equation of RPC.

Equation	Applicable Object	Reference
y=0.0338x	RPC	Qu, W. et al. [52]
$y=ax+\left(5-4a\right)x^{4}+\left(3a-4\right)x^{5}$	RPC	Ma, Y. [53]
$\sigma =400\varepsilon \left(1-75\varepsilon \right)f_{c}$	CFRPC	Ke, K. et al. [36]
$y=1.2x+0.2x^{4}-0.4x^{5}$	SFRPC	Zhou, C. [54]
$y=ax+\left(6-5a\right)x^{5}+\left(4a-5\right)x^{6}$	BFRPC	Shen, T. [55]

## Data Availability

The data presented in this study are available on request from the corresponding author.
